# Identifying dendritic processing in a [Filter]-[Hodgkin Huxley] circuit

**DOI:** 10.1186/1471-2202-12-S1-P306

**Published:** 2011-07-18

**Authors:** Aurel A Lazar, Yevgeniy B Slutskiy

**Affiliations:** 1 Department of Electrical Engineering, Columbia University, New York, NY 10025, USA

## 

The nature of encoding and processing of sensory information in the visual, auditory and olfactory systems has been extensively investigated in the systems neuroscience literature. Many phenomenological [1] as well as mechanistic [2] models have been proposed to characterize and clarify the representation of sensory information on the level of single neurons. In [3] we presented a novel methodology for identifying dendritic processing in phenomenological neural circuit models in which the time-domain linear processing takes place in the dendritic tree and the resulting aggregate dendritic current is encoded in the spike domain by a spiking neuron. In block-diagram form, these neural circuit models are of the [Filter]-[Spiking Neuron] type and as such represent a fundamental departure from the standard Linear-Nonlinear-Poisson (LNP) model that has been used to characterize neurons in many sensory systems, including vision [4], audition [1] and olfaction [5]. While the LNP model also includes a linear processing stage, it describes spike generation using an inhomogeneous Poisson process. In contrast, the [Filter]-[Spiking Neuron] model incorporates the temporal dynamics of spike generation and allows one to consider biophysically inspired spike generators.

Here we extend the dendritic identification results of [3] to spiking neurons with random parameters and consider a more general model of dendritic processing. As before, we assume that input stimuli belong to the space of bandlimited functions, but now formulate the filter identification problem using the theory of smoothing splines. This alternative formulation allows us to take into account noise sources inherently present in biological neurons and makes our methods amenable to modeling neural circuits that arise in systems neuroscience. First, we construct an optimal estimate of the dendritic processing filter in a neural circuit consisting of a filter in cascade with an ideal integrate-and-fire (IAF) neuron with random threshold (RT), also abbreviated as the [Filter]-[Ideal IAF/RT] circuit. Second, we demonstrate that our basic method can be extended to identify the dendritic processing filter in cascade with a Hodgkin-Huxley (HH) neuron with stochastic conductances (SC). We then investigate two different ways in which a stimulus can be coupled to a HH neuron: multiplicative coupling ([Filter]-[HH/SC/MC] circuit) [6] and additive coupling ([Filter]-[HH/SC/AC] circuit). We also show that using conditional phase response curves (cPRCs) [7] it is possible to identify the dendritic processing filter using both weak and strong input stimuli. Third, we demonstrate that the presented methodology is very general and can be employed by system neuroscientists to model neural circuits using a (biophysical) neuron model of their choice. Specifically, we show that the above identification methods can be readily applied to any neural (nonlinear dynamical) system with a limit cycle, including FitzHugh-Nagumo, Morris-Lecar and I_Na,p_ + I_K_ models.

## References

[B1] Sean Slee J. Matthew Higgs H. Adrienne Fairhall L. William Spain J. Two-dimensional time coding in the auditory brainstem The Journal of Neuroscience 2005 25 43 9978 9988 10.1523/JNEUROSCI.2666-05.2005 16251446 PMC6725565

[B2] Zhuoyi Song Daniel Coca Stephen Billings Marten Postma Roger Hardie C. Mikko Juusola Biophysical Modeling of a Drosophila Photoreceptor Lecture Notes In Computer Science, volume 5863 of Proceedings of the 16th International Conference on Neural Information Processing: Part I 2009 Springer-Verlag 57 71

[B3] Aurel Lazar A. Yevgeniy Slutskiy B. J. Lafferty and C. K. I. Williams and J. Shawe-Taylor and R.S. Zemel and A. Culotta Identifying dendritic processing Advances in Neural Information Processing Systems 23 2010

[B4] Chichilnisky EJ A simple white noise analysis of neuronal light responses Network: Computation in Neural Systems 2001 12 199 213 11405422

[B5] Anmo Kim J. Aurel Lazar A. Yevgeniy Slutskiy B. System identification of Drosophila olfactory sensory neurons Journal of Computational Neuroscience 2010 30 1 10.1007/s10827-010-0265-0 PMC3736744 20730480

[B6] Aurel Lazar A. Population encoding with Hodgkin-Huxley neurons IEEE Transactions on Information Theory 2010 56 2 821 837 10.1109/TIT.2009.2037040 PMC3816091 24194625

[B7] Anmo Kim J. Aurel Lazar A. Recovery of stimuli encoded with a Hodgkin-Huxley neuron using conditional PRCs Phase Response Curves in Neuroscience 2011 Springer available at http://www.bionet.ee.columbia.edu/publications.html

